# Identity Development and Maladaptive Personality Traits in Young Refugees and First- and Second-Generation Migrants

**DOI:** 10.3389/fpsyt.2021.798152

**Published:** 2022-01-21

**Authors:** Max Zettl, Zeynep Akin, Sarah Back, Svenja Taubner, Kirstin Goth, Catharina Zehetmair, Christoph Nikendei, Katja Bertsch

**Affiliations:** ^1^Center for Psychosocial Medicine, Institute for Psychosocial Prevention, Medical Faculty, University Hospital Heidelberg, Heidelberg, Germany; ^2^Department of Psychology, Ludwig-Maximilians-University Munich, Munich, Germany; ^3^Department of Child and Adolescent Psychiatric Research, Psychiatric University Clinics (UPK), Basel, Switzerland; ^4^Department of General Internal Medicine and Psychosomatics, University Hospital Heidelberg, Heidelberg, Germany; ^5^Department of General Psychiatry, Medical Faculty, Center for Psychosocial Medicine, University Hospital Heidelberg, Heidelberg, Germany

**Keywords:** identity, maladaptive traits, personality, refugees, migration

## Abstract

Refugees are often exposed to a variety of stressors and traumatic events, posing a significant risk for the development of mental disorders. Young refugees may be particularly at risk because adverse life events affect identity formation, a developmental task that is typically expected in adolescence and emerging adulthood. Trauma and cultural changes may alter identity development, potentially leading to identity diffusion, a core concept of personality disorders. However, previous research on personality pathology among refugees is scarce. In this study, we examine identity development and maladaptive personality traits in young refugees and migrants. Refugees from 22 countries of origin were recruited in a German reception center (*n* = 120) and a group of adults with a migration background in first- or second generation was obtained via web-based recruitment (*n* = 281). Identity development was measured using the Assessment of Identity Development in Adolescence – Short Form. Maladaptive personality traits were assessed with the Personality Inventory for DSM-5-Brief Form. Group differences between refugees and migrants regarding identity development and trait expression were investigated using *t*-tests. The relationship between the two measures and their corresponding subscales was examined by means of correlation analyses. Refugees reported significantly higher levels of identity diffusion, negative affectivity, detachment, antagonism, and disinhibition compared to migrants. No significant differences were found for psychoticism. Correlation analyses revealed low to moderate positive associations between identity diffusion and maladaptive trait expression. Possible implications for early phase of resettlement, preventive psychiatric care and further research questions are discussed.

## Introduction

According to the United Nations High Commissioner for Refugees' 2020 Global Trends Forced Displacement report (1), 82.4 million people have been forcibly displaced worldwide, a number which is approximately twice as high as 10 years ago and the highest record to date. Being forced to flee their homes due to persecution or violent conflicts, refugees and asylum seekers frequently experience pre- and peri-flight traumatic events (2–4) and high mental health needs, including increased rates of posttraumatic stress disorder (PTSD) and depression (5, 6). However, prevalence estimates vary significantly between studies (7, 8) and there is a lack of studies on the full spectrum of mental disorders (5, 9). Although trauma is an important etiologic factor in the development of personality disorders (PDs), which demonstrate substantial comorbidity rates with PTSD (10–12), current research on personality pathology in refugees is scarce. Moreover, refugees face a variety of post-migration stressors during the resettlement process (13), such as awaiting the decision on the asylum application, living in reception centers, separation from the family, and adjusting to life in a new host country. For young refugees, this phase may be particularly challenging, as identity formation is a key developmental task during adolescence and emerging adulthood (14, 15). Failure to develop a continuous and coherent sense of self and others is a hallmark of personality pathology (16–18), however, identity development and its relationship to maladaptive personality traits has not yet been studied in refugees and asylum seekers. As mental health difficulties are associated with poorer integration (19), it is crucial to evaluate refugees and asylum seekers' mental health burden and promote preventive strategies to improve mental health. The present study aims to address this gap in the literature by examining identity development and maladaptive personality traits in a sample of young refugees compared to first- and second-generation migrants.

### Identity Development

The historical origins of the concept of identity reach back almost 100 years (20) and is related to psychodynamic (21) and social-cognitive theory (22). Erikson's stages of psychosocial development (15) proposed a broad definition of identity as a “[…] fundamental organizing principal […]” [(23), p. 98], predominantly forming during adolescence that provides continuity of self but also a sense of uniqueness to distinguish between self and others (15, 23). Nowadays, in Western, industrialized countries, identity exploration is thought to expand into emerging adulthood (i.e., 18–29 years of age) (14, 24). Several determinants influence identity development, including individual (e.g., age and sex), social (e.g., family and peers), and cultural factors (e.g., collectivistic and individualistic societies) (25, 26). In addition, critical life events such as job loss, migration, or health impairment are associated with identity changes in adolescence and adulthood (27–29). However, if a clear and coherent sense of identity is not reached, non-integrated representations of self and significant others are “[…] split into an idealized and persecutory segment […]” [(16), p. 977], which Kernberg (16) describes as the syndrome of identity diffusion and a hallmark of personality pathology. Although identity impairment has previously been a diagnostic criterion mainly for borderline PD (30), the concept has been re-introduced in recent years to the dimensional PD classification systems in DSM-5 (31) and ICD-11 (32). The Alternative Model for Personality Disorders (AMPD) in DSM-5's Section III (31), designed to address the shortcomings of the categorical PD taxonomy (33), introduced impairments in self-functioning (identity and self-direction) and interpersonal functioning (empathy and intimacy) (31, 34) as central diagnostic criteria for all PD types (31). Moreover, ICD-11 (35) replaces traditional PD categories and defines personality pathology “by problems in functioning of aspects of the self (identity, self-worth, accuracy of self-view, self-direction), and/or interpersonal dysfunction.”

### Maladaptive Personality Traits

With DSM-5's alternative model (31) and the eleventh version of the ICD (35), trait-based assessment of PD is increasingly shaping the classification of personality pathology. The DSM-5 PD workgroup introduced an empirically based model of pathological personality traits for PD assessment in Criterion B in the AMPD (36, 37). According to this model, personality dimensions are organized in five higher order domains (negative affectivity, detachment, antagonism, disinhibition, and psychoticism) that represent maladaptive variants of the Five Factor Model of normative personality (31, 38–40). Each domain comprises a set of subordinate trait facets (e.g., the domain antagonism is split up into the facets manipulativeness, deceitfulness, grandiosity, attention seeking, callousness, and hostility) (31). Trait constructs not only provide a description of a person's personality style, but also demonstrate important clinical utility by predicting important diagnostic variables [e.g., hospitalization and suicide attempts; (41, 42)] and informing clinical decision making [e.g., intervention planning; (43–46)]. The occurrence of maladaptive trait domains can be demonstrated as early as in childhood (47) and remain relatively stable across different developmental stages (48–51), even over the course of treatment (52). Identity and other elements of personality function, however, represent more dynamic aspects of personality pathology (53). The maladaptive personality dimensions can be assessed using structured interviews (54) and questionnaires in self-report and informant-form, including the broadly validated Personality Inventory for DSM-5 (31, 37, 55).

### Identity Development, Maladaptive Traits, and Migration Experiences

The experience of having to flee one's home or being displaced is a traumatic event that results in a radical change in familial, relational, social, and cultural life. However, little is known about how this experience affects the identity development of young refugees and asylum seekers, although preliminary studies suggest that the resettlement process is associated with identity problems (56, 57). Therefore, the first objective of this study is (a) to examine identity development among young refugees. To consider cultural aspects in identity development, a group of first- and second-generation migrants with no refugee experience was obtained as a control group.

Systematic reviews and meta-analyses document the high prevalence of mental disorders among refugees and asylum seekers (5, 7, 58). However, PDs have rarely been assessed in previous studies. A study by Latzman et al. (59) on callous and unemotional traits demonstrated that immigrant adolescents who had lived in a refugee camp prior to migration reported higher uncaring trait levels than migrants with no camp experience, which in turn was related to lower levels of agreeableness and openness. Therefore, there is a need to identify the diverse range of psychopathological symptoms typically found in refugee and asylum seeker populations. The second goal of this study is (b) to investigate maladaptive traits in refugees, a population exposed to a variety of stressors (e.g., living in a reception center and awaiting asylum decision), compared to migrants who have been born or living in the new host society for several years.

Identity processes act as a dynamic force in the development of personality over time (60, 61) that promote personality consistency (62). Impaired identity development has been associated with higher neuroticism (63, 64). However, the relationship between identity development and traits have been studied primarily in adolescent and student populations (65), and none of the previous work has addressed maladaptive traits among refugees and asylum seekers. Therefore, the third goal of this study is (c) to examine the relationship between identity development and maladaptive traits according to Criterion B of the AMPD.

## Methods

### Materials and Procedures

The present study is based on the ethical principles of the Declaration of Helsinki (66) and was approved by the Ethics Committee of the Medical Faculty of the University of Heidelberg, Germany (S-636/2014) and the Ethics Committee of the Faculty of Psychology and Educational Sciences of Ludwig-Maximilians-University Munich (35_a_2019). Analyses are based on a sample of young refugees (*n* = 120) and a community sample of adults (*n* = 281) with a migration background in first- or second generation (i.e., individuals born outside of Germany or with at least one foreign-born parent).

Refugees were recruited at a German registration and reception center between May and October 2019. The survey was part of a larger project to develop an instrument to assess mental burden in refugees and asylum seekers. General exclusion criteria comprised (1) underage (<18 years) and (2) illiteracy prior to study. After providing written informed consent, participants completed a set of digitally provided questionnaires.

Young adults with an EU or a non-EU migration background were recruited during January and March 2020. The study was advertised on social media and accessible through the SoSci-Survey-portal (www.soscisurvey.de). General inclusion criteria comprised (1) age ≥ 18, (2) a migrant background in the first- or second-generation, and (3) sufficient knowledge of the German language as the questionnaires for this group were provided in German only. Electronic informed consent forms were obtained from all participants. Participants did not receive financial reimbursement but were able to take part in a raffle in which ten 25€-Amazon vouchers were drawn.

#### Sample of Young Refugees

A total of 120 young adult refugees (59 female, *M*_age_ = 28.13, *SD* = 5.70, range: 18–40 years) from 22 countries of origin participated in the study, including Nigeria (30%), Iran (20%), Turkey (10%), Iraq (8.3%), Syria (5%), Afghanistan (3.3%), and Tunisia (3.3%). The questionnaires employed were available in English (34.2%), Persian (25%), Arabic (24.2%), Turkish (10.8%), Croatian (4.2%), French (0.8%), and German (0.8%). When asked about one or more reasons for their departure, refugees indicated that they had experienced a threat to their family (35%), war (20%), political persecution (19.2%), torture (18.3%), discrimination (16.7%), loss of a family member (13.3%), abuse and/or rape (13.3%), and other reasons (<10%). In terms of religious affiliation, 49.2% of respondents reported being Muslim, followed by Christianity (40%) and other religious beliefs (e.g., Atheism, Buddhism, Hinduism, Judaism). Participants varied significantly in terms of their educational background with 37.5% reporting to have attended school, 25.8% holding a high school diploma, 25.8% holding a University degree, and 10.8% reporting no formal schooling at all. In a self-report form, 28.3% reported suffering from mental health problems and 18.3% were taking any psychotropic medication at the time of the study.

#### Sample of First- and Second-Generation Migrants

Two hundred eighty-one young adults (241 female, 1 diverse, *M*_age_ = 23.29, *SD* = 4.37, range: 18–40 years) with a first- or second-generation migration background enrolled in this study. Regarding foreign origin, 16.4% of respondents indicated being born outside of Germany (i.e., first-generation migrants), while the vast majority (83.6%) were native-born German citizens with at least one foreign parent (i.e., second-generation migration). Most participants had lived in Germany for many years: 30.4% of first-generation migrants and 74% of second-generation migrants reported that the date of migration was more than 20 years ago, compared to 10 or fewer years for 4.4 and 39.1%, respectively. First-generation migrants originated from 22 countries, while the parents of second-generation migrants were mostly from Turkey (43.7%), Bosnia-Herzegovina (10.8%), and Afghanistan (8.1%). 92.5% of respondents reported being Muslim, followed by Christianity (3.6%) and other religious affiliations (e.g., Atheism, Buddhism, Hinduism, Judaism). Participants had a high level of education: 64.1% had a high school diploma and another 34.9% held a University degree. Two participants reported having a primary school diploma. At the time of participation, 65.1% were enrolled as students, 15.3% employed, 5.7% trainees, 4.6% pupils, 3.9% unemployed, and 2.8% self-employed; 2.5% indicated other occupational statuses.

### Measures

#### Assessment of Identity Development in Adolescence – Short Form (AIDA Short)

Identity development was measured using the AIDA Short (especially created research version by the original authors for supporting this study), a 23-item self-report inventory assessing impairments in identity in adolescents and young adults. The AIDA Short was developed from the original 58-item AIDA (67), a comprehensive measure of healthy and disturbed identity development in terms of personality functioning (Criterion A) that integrates approaches from both psychoanalytic and social-cognitive psychology (68, 69). Each item is answered on a 5-point Likert-scale ranging from 0 (*no*) to 4 (*yes*). The version AIDA Short is equivalent to the scale “Identity” of the questionnaire LoPF-Q 12-18 (70) to assess the full spectrum of personality functioning with the four domains identity, self-direction, empathy, and intimacy. It was developed based on empirical item selection in school and clinic samples and showed good scale reliability (71). The identity total score also differed at a highly significant level and with a relevant effect size of d = 2.0 standard deviations between the general population and a subsample of *N* = 96 patients diagnosed with PD (SCID-2) as a sign of excellent clinical validity. Compared to the original AIDA with 58 items, the scoring of the AIDA Short provides a total scale (identity integration vs. identity diffusion), as well as two subscales, namely discontinuity and incoherence. The discontinuity scale assesses lack of identity-consolidating perspectives, roles, and emotional self-experience, whereas the incoherence scale reflects inconsistent self-images, lack of autonomy, and diffuse representations [see (68, 69) for a detailed description]. The scales are coded toward the degree of identity impairment. The AIDA full version is available in various language versions, is freely available for research projects at the project website (https://academic-tests.com), and its psychometric properties have documented in several studies and populations (68, 69, 72–75). In the total sample, internal consistencies of two subscales were acceptable to good, with Cronbach's α ranging from 0.73 for identity discontinuity to 0.83 for identity incoherence. For the total scale, internal consistency was good with Cronbach's α = 0.87.

#### Personality Inventory for DSM-5 – Brief Form (PID-5-BF)

The PID-5-BF is a 25-item self-report questionnaire assessing maladaptive personality traits according to Criterion B of the AMPD (APA, 2013). Developed from the 220-item pool of the original PID-5 (37), the PID-5-BF provides a brief screening measure of the AMPD's higher order trait domains of negative affectivity, detachment, antagonism, disinhibition, and psychoticism (APA, 2013). Each domain is measured by 5 items, which are rated on a 4-point Likert-scale ranging from 0 (*very false or often false*) to 3 (*very true or often true*). The scoring procedure provides mean scores for each domains as well as an overall mean score, coded toward personality pathology (31). The full version of the PID-5 has been used in numerous studies and demonstrates adequate validity and reliability (55), fewer studies, on the other hand, have been devoted to the psychometric properties of the 25-item version (76). In this study, internal consistencies of the PID-5 trait scales varied substantially, with Cronbach's α ranging from 0.61 for antagonism to 0.76 for psychoticism. For the total scale, internal consistency was good with Cronbach's α = 0.88.

### Statistical Analyses

Because data collection was done electronically employing a forced-choice format, the data set did not contain any missing values. All statistical analyses were performed using JASP (version 0.14.1.0). The significance level was set at *p* < 0.05, two-tailed. Effect sizes are interpreted according to Cohen (77): small (d = 0.2), medium (d = 0.5), and large (d = 0.8) effect. Descriptive statistics for the AIDA Short and the PID-5-BF were obtained separately for the refugee and migrant sample. Preliminary analyses examined associations between the AIDA Short, PID-5-BF, and age and sex.

For our first and second research question, i.e., whether refugees and first- and second-generation migrants show differences in the expression of identity development and maladaptive traits, we conducted independent samples Welch's *t*-tests. Bonferroni correction was applied to adjust for multiple comparisons. For our third research question, i.e., whether identity development is related to maladaptive personality traits in refugees and migrants, we ran Spearman correlation analyses. Correlation coefficients were calculated separately by group. Statistically significant correlations between the AIDA Short and the PID-5-BF were then transformed using Fisher's *r* to *z* transformation to test for between-group differences.

## Results

Descriptive statistics of the AIDA Short and the PID-5-BF in the refugee and migrant samples are provided in Table 1. Exploratory analyses in the total sample are available in the electronic Supplementary Material.

**Table 1:** Descriptive statistics, reliability, and group differences between the refugee and migrant sample.

**Scale**	**Refugee sample (*****n*** **=** **120)**					**Migrant sample (*****n*** **=** **281)**					**Welch's** ***t*****-test**			
	***M***	***SD***	***Skew***	**Kurt**	**α**	***M***	***SD***	***Skew***	**Kurt**	**α**	***t***	***df***	***p***	**Cohen's *d***
AIDA Short total	36.35	17.71	0.47	−0.41	0.85	29.78	13.87	0.53	−0.22	0.88	3.62	184.10	<0.01	0.41
AIDA Short discontinuity	14.09	8.64	0.95	0.77	0.72	12.92	6.28	0.58	0.29	0.75	1.34	175.05	1	0.15
AIDA Short incoherence	22.26	11.62	0.18	−0.96	0.82	16.86	8.63	0.56	−0.30	0.83	4.58	177.38	<0.001	0.53
PID-5-BF total	1.19	0.55	0.19	−0.69	0.87	0.94	0.42	0.45	0.25	0.87	4.42	181.02	<0.001	0.51
PID-5-BF negative affectivity	1.59	0.79	−0.02	−0.82	0.71	1.29	0.61	0.10	−0.47	0.67	3.74	182.04	<0.01	0.43
PID-5-BF detachment	1.25	0.77	0.26	−0.62	0.66	0.94	0.58	0.59	0.22	0.67	3.91	180.19	<0.01	0.45
PID-5-BF antagonism	0.78	0.53	1.16	2.48	0.52	0.48	0.47	1.39	2.64	0.69	5.36	203.71	<0.001	0.60
PID-5-BF disinhibition	1.21	0.70	0.34	−0.28	0.62	0.95	0.56	0.46	−0.16	0.71	3.64	187.70	<0.01	0.41
PID-5-BF psychoticism	1.11	0.84	0.32	−0.88	0.78	1.03	0.65	0.47	−0.39	0.75	0.86	183.10	1	0.10

*AIDA Short, Assessment of Identity Development in Adolescence – Short Form; PID-5-BF, Personality Inventory for DSM-5 – Brief Form. P-values are adjusted with Bonferroni correction for multiple comparisons*.

### Do Refugees and First- and Second-Generation Migrants Show Differences in the Expression of Identity Development?

Test statistics and effect sizes are listed in Table 1. Refugees showed significantly higher levels of identity diffusion compared to migrants. Cohen's effect size value suggested a small to medium effect for the total score of the AIDA Short. Regarding the two subscales of the AIDA Short, the results were mixed: While refugees demonstrated significantly higher levels of identity incoherence, resulting in a medium-sized effect, no mean difference was found regarding identity discontinuity.

### Do Refugees and First- and Second-Generation Migrants Show Differences in the Expression of Maladaptive Personality Traits?

An independent samples *t*-test revealed that refugees demonstrated significantly higher overall expression of maladaptive personality traits than migrants with a medium effect size. Regarding the DSM-5 trait domains, refugees scored significantly higher on negative affectivity, detachment, antagonism, and disinhibition with medium effect sizes. However, no significant difference was found between the two groups for psychoticism.

### Is Identity Development Related to Maladaptive Personality Traits in Refugees and First- and Second-Generation Migrants?

Bivariate correlations among the AIDA Short scales and the DSM-5 trait domains are displayed in Table 2. The AIDA Short total score was significantly positively associated with all PID-5-BF scales, in both refugees (*r* = 0.36–0.66) and migrants (*r* = 0.27–0.55). In the refugee group, the strongest associations were found between identity diffusion and detachment (*r* = 0.66) as well as psychoticism (*r* = 0.66), the lowest between identity diffusion and antagonism (*r* = 0.36). In the migrant sample, the highest correlation was found between identity diffusion and negative affectivity (*r* = 0.65), the lowest for antagonism (*r* = 0.27). The two subscales of the AIDA Short were also significantly associated with all DSM-5 trait domains, in refugees (*r* = 0.24–0.65) and migrants (*r* = 0.20–0.67). Using Fisher's *r* to *z* transformation and a two-tailed test of significance, no significant differences were found in the correlations between identity diffusion and trait domains. Only the AIDA Short identity discontinuity subscale demonstrated a significantly higher association with detachment in refugees (*r* = 0.65) than migrants (*r* = 0.50).

**Table 2:** Intercorrelations between the AIDA Short and the PID-5-BF in the refugee (below the diagonal, *n* = 120) and the migrant sample (above the diagonal, *n* = 281).

**Scale**	**1**	**2**	**3**	**4**	**5**	**6**	**7**	**8**
1. AIDA Short total	–	0.90^***^	0.95^***^	0.65^***^	0.54^***^	0.27^***^	0.50^***^	0.55^***^
2. AIDA Short discontinuity	0.81^***^	–	0.72^***^	0.52^***^	0.50^***^	0.20^***^	0.45^***^	0.44^***^
3. AIDA Short incoherence	0.91^***^	0.53^***^	–	0.67^***^	0.51^***^	0.28^***^	0.46^***^	0.56^***^
4. PID-5-BF negative affectivity	0.64^***^	0.43^***^	0.64^***^	–	0.40^***^	0.33^***^	0.45^***^	0.49^***^
5. PID-5-BF detachment	0.66^***^	0.65^***^	0.54^***^	0.58^***^	–	0.26^***^	0.32^***^	0.48^***^
6. PID-5-BF antagonism	0.36^***^	0.24^**^	0.37^***^	0.36^***^	0.35^***^	–	0.40^***^	0.39^***^
7. PID-5-BF disinhibition	0.44^***^	0.39^***^	0.38^***^	0.45^***^	0.46^***^	0.34^***^	–	0.56^***^
8. PID-5-BF psychoticism	0.66^***^	0.48^***^	0.64^***^	0.64^***^	0.59^***^	0.32^***^	0.46^***^	–

*AIDA Short, Assessment of Identity Development in Adolescence – Short Form; PID-5-BF, Personality Inventory for DSM-5 – Brief Form*.

** *p < 0.01*,

*** *p < 0.001. Significant between-group differences for the AIDA Short and PID-5-BF are in bold*.

## Discussion

This study investigated identity development and maladaptive traits in refugees living in a registration and reception center and first- and second-generation migrants who were born in Germany or had lived in the host society for several years.

The first objective of this study was to examine whether the two groups exhibit differences in the extent of identity development. Participants from the refugee sample reported significantly higher levels of identity diffusion as measured by the total scale of the AIDA Short. This result is consistent with previous research showing that refugees face identity-related challenges and problems (56). However, the results were less decisive at the substructure of identity: Refugees yielded higher scores on the incoherence subscale, but the two groups did not differ regarding the discontinuity scale. Thus, refugee identity in the present sample seems to be less pronounced only in terms of coherence of identity integration, i.e., being often confronted with contradictions, over-identification, and superficial, diffuse representations regarding the clarity of their self-definition (68). However, no differences were found between refugees and migrants regarding identity continuity, that is, the capacity to dedicate oneself to long-term goals, to internalize stable moral and inner values, and to have a sense of subjective self-sameness (68). In contrast, other research has also found significant differences with respect to this identity component: Ertorer (56) studied identity problems among Karen refugees from Burma and found lower identity continuity compared to non-migrants from the host society. However, the two studies applied different methods in measuring identity and the present study used a migrant sample for normative comparison, whereas as Ertorer (56) recruited non-migrant individuals. The present findings suggest that refugees are more likely to experience conflicting or ambivalent self-images. This finding could be explained by the fact that refugees and asylum seekers are repeatedly confronted with discrepancies between their self-image and the cultural environment of the host society (e.g., social norms, cultural values, and language) through forced migration, resettlement, and acculturation.

The second aim of this study was to examine whether refugees and first- and second-generation migrants differ in the expression of maladaptive personality traits. Refugees reported higher overall expression of maladaptive personality traits and had higher scores in four of the five DSM-5 personality domains, while no group difference was observed for psychoticism. The refugee sample was recruited in a reception center where asylum seekers are required to stay until a decision is made on their asylum application. Prolonged stress in arrival and reception centers, without certainty about the future, can trigger intense experiences of negative emotions that may manifest in higher levels of negative affectivity. Traumatic experiences and the high prevalence of PTSD among refugees may contribute to the frequent and intense experience of negative emotions: Doolan et al. (78) investigated emotion regulation in traumatized refugees, demonstrating that PTSD symptoms are associated with emotion regulation difficulties, in particular a lack of access to emotion regulation strategies, and a lack of emotional clarity.

Refugees also reported higher levels of detachment, which could be a coping mechanism to deal with negative experiences during migration and the stressful environment in a reception center. To protect themselves from these highly arousing situations, they may develop a tendency to avoid interpersonal interactions and express restricted affective experiences. This is in line with the aforementioned study by Latzmann et al. (59) that adolescent migrants who had lived in a refugee camp reported higher uncaring trait levels.

Furthermore, refugees displayed higher levels of antagonism and disinhibition. Antagonism (e.g., manipulativeness, callousness, deceitfulness, and hostility) and disinhibition (e.g., risk taking, impulsivity and irresponsibility) are prominent characteristic traits of antisocial PD. However, little is known about the etiology of this disorder, nor about its prevalence among refugees as it is often not assessed [e.g., (79)]. Because the migrant group in our study was predominantly female, it seems likely that these results reflect sex differences in levels of antagonism and disinhibition (80) rather than trait differences between refugees and migrants.

A systematic review by Brand et al. (81) found that refugees have an increased risk for developing schizophrenia and associated non-affective psychoses, compared to non-refugee migrants and native populations. In contrast, the present study found no difference between refugees and migrants regarding levels of psychoticism. However, it should be noted that psychoticism in the AMPD, defined by unusual beliefs and experiences, eccentricity, and cognitive and perceptual dysregulation (31), demonstrates only moderate correlations with psychotic symptoms (82). Moreover, a recent meta-analysis by Blackmore et al. (5) reported comparable rates of psychosis in refugees and the general population.

The third objective of this study was to examine the relationship between identity development and maladaptive personality traits. In both samples, significant positive association between identity diffusion and maladaptive trait severity were observed. Regarding the hierarchical structure of personality traits, impairments in identity were somewhat more strongly associated with the internalizing spectrum of personality pathology (negative affectivity and detachment) than with the externalizing component (antagonism and disinhibition). Exploratory analyses of differences in correlation coefficients between the groups revealed a consistent pattern, with only identity discontinuity and detachment showing a slightly stronger association in the refugee sample. Therefore, the present results replicate previous findings on identity and its relation to the normative Big Five personality traits [e.g., reduced identity development is associated with higher neuroticism (63, 64)] and extend it to a multi-ethnic mixed sample. However, no specific patterns between constituents of identity and individual traits can be established in either of the present samples, as traits, among other factors, also show significant intercorrelations.

### Implications for Early Phase of Resettlement and Preventive Psychiatric Care

Diagnosing personality pathology is considered difficult (83), and PD assessment by clinical interviews is often impractical in studies with time constraints (79). However, PDs are prevalent globally (84) and associated with greatly reduced life expectancy (85). The assessment of personality pathology in adolescents and young adults is particularly important because PDs usually develop during this developmental period (86). Early detection of personality impairments and promotion of appropriate psychosocial interventions for this at-risk population soon after their arrival, may counteract the manifestation of a PD. However, although there is a high need for care, refugees' access to psychosocial services is often inadequate (87). Therefore, in addition to identifying mental health problems, it is equally important to overcome corresponding barriers (e.g., legal and language barriers) (88, 89). Our results may be interpreted in the direction that supporting identity development in refugees might be a successful strategy to promote resilience. This may be in addition to classic mental health care by allowing refugees to share their cultural heritages with the hosting cultures, exchange and discuss cultural norms, and by relieving them of the impression that they would need to give up cultural identity if they want to remain in the hosting country.

### Limitations and Future Directions

The present study had some limitations that should be considered in the interpretation of the results. First, there was no standardized assessment of mental health problems or disorders, so their effects on identity functioning and personality traits could not be considered in the analyses. Future studies should therefore include a structured assessment of common mental disorders among refugees and asylum seekers. Second, the generalizability of our results is limited to comparisons between refugees and first- and second-generation migrants, as no individuals without any migration experience were included in this study. This is particularly relevant considering the immigrant paradox: studies suggest that immigrants, especially when they move from low-income to high-income countries, demonstrate better mental health than their counterparts without migration experience (90–92). Initial studies suggest that this effect also applies to personality pathology, as immigrants also have a lower risk of developing a PD than native-born citizens (91, 93). Therefore, future studies of personality in refugees should include individuals without any migration experience. Furthermore, only German-speaking first- and second-generation immigrants were included in this study, limiting the generalizability of the results. Third, refugees participated in the study briefly after their arrival in Germany, and little is known to date about the immediate impact of refugee experiences on psychological well-being. In addition, situational factors (e.g., living in a reception center and waiting for the asylum decision) may represent a potential bias in self-assessment of identity and personality and underscore the need for longitudinal studies. Fourth, methodological aspects should be considered in the operationalization of identity and personality: The AIDA Short and AIDA are designed to assess identity development in adolescents (12–18 years). Age-relevant differences in the assessment of identity should therefore be considered in the selection of instruments in future studies. Meanwhile a special version AIDA 19+ for young adults is established and only 5 items had to be adjusted very slightly to also fit for older probands (94). However, this age-adapted version of the AIDA is currently only available in English and German and was therefore not suitable for this study. Furthermore, the PID-5-BF is only a screening instrument and does not allow the diagnosis of PD or the assessment of lower-order personality facets. In addition, its psychometric properties are less well-documented and internal consistency of the PID-5-BF scales varied significantly in the present study. ICD-11 also introduces the domain of anancasm in place of psychoticism into its trait-model (35). Therefore, future studies should employ new instruments such as the PID-5-BF+ (95) that provide assessment of maladaptive traits from the perspective of DSM-5 and ICD-11.

To summarize, this cross-sectional study demonstrates that refugees experience higher levels of identity impairment and maladaptive personality traits compared to first- and second-generation migrants. Furthermore, identity diffusion is significantly related to maladaptive trait expression in both samples.

## Data Availability Statement

The raw data supporting the conclusions of this article will be made available by the authors, without undue reservation.

## Ethics Statement

This study was approved by the Ethics Committee of the Medical Faculty of the University of Heidelberg, Germany (S-636/2014) and the Ethics Committee of the Faculty of Psychology and Educational Sciences of Ludwig-Maximilians-University Munich (35_a_2019). The patients/participants provided their written informed consent to participate in this study.

## Author Contributions

MZ has analyzed the data and written the manuscript. ZA has recruited participants, collected data, and supported data analyses and manuscript writing. SB, KG, CN, and CZ have supported manuscript writing. ST and KB have received funding for the study, designed the study, supervised data collection, and supported data analyses and manuscript writing. All authors have read, reviewed, and approved the final version of the manuscript.

## Funding

This study was supported by a grant from the Center for Advanced Study Marsilius Kolleg of Heidelberg University awarded to ST and KB.

## Conflict of Interest

The authors declare that the research was conducted in the absence of any commercial or financial relationships that could be construed as a potential conflict of interest.

## Publisher's Note

All claims expressed in this article are solely those of the authors and do not necessarily represent those of their affiliated organizations, or those of the publisher, the editors and the reviewers. Any product that may be evaluated in this article, or claim that may be made by its manufacturer, is not guaranteed or endorsed by the publisher.
