# Supplementary material for: Identity Development and Maladaptive Personality Traits in Young Refugees and First- and Second-Generation Migrants
Source: Front Psychiatry. 2022 Jan 21;12:798152. doi: 10.3389/fpsyt.2021.798152 (PMC8813733; doi:10.3389/fpsyt.2021.798152)
Supplement: Supplementary file 1 [file Data_Sheet_1.docx]

Supplementary Material

Exploratory analyses in the total sample revealed that increased age was associated with lower levels of psychoticism, *r*(399)=-.15, *p*<.01. In addition, age was negatively correlated with identity discontinuity, *r*(399)=-.15, *p*<.01. However, both associations were weak. Considering sex, men (*M*=.81, *SD*=.59) displayed higher levels of antagonism compared to women (*M*=.49, *SD*=.45), *t*(140.305)=4.862, *p*<.001. Moreover, men (*M*=1.15, *SD*=.60) demonstrated higher scores for disinhibition than women (*M* =.99, *SD*=.62), *t*(172.683)=2.234, *p*=<.05. This sex difference was also found for the total scale of the PID-5-BF, with men (*M*=1.13, *SD*=.52) showing higher levels of maladaptive personality traits compared to women (*M*=.98, *SD*=.46), *t*(153.043)=2.624, *p*=<.05. First- and second-generation migrants did not differ with respect to identity development or maladaptive personality traits (all *p*>.05).
